# Lung cancer patients have the highest malignancy-associated suicide rate in USA: a population-based analysis

**DOI:** 10.3332/ecancer.2018.859

**Published:** 2018-08-16

**Authors:** Mohamed Rahouma, Mohamed Kamel, Ahmed Abouarab, Ihab Eldessouki, Abu Nasar, Sebron Harrison, Benjamin Lee, Eugene Shostak, John Morris, Brendon Stiles, Nasser K Altorki, Jeffrey L Port

**Affiliations:** 1 Weill Cornell Medicine, New York Presbyterian Hospital, New York, NY 14853 USA; 2 Vontz Molecular Center, Hemato-oncology Department, University of Cincinnati, Cincinnati, OH 45220, USA

**Keywords:** standardised mortality ratio (SMR), SEER database, suicide, lung cancer, psychological support

## Abstract

**Purpose:**

Previous studies have reported that psychological and social distresses associated with a cancer diagnosis have led to an increase in suicides compared to the general population. We sought to explore lung cancer-associated suicide rates in a large national database compared to the general population, and to the three most prevalent non-skin cancers [breast, prostate and colorectal cancer (CRC)].

**Methods:**

The Surveillance, Epidemiology and End Results (SEER) database (1973–2013) was retrospectively reviewed to identify cancer-associated suicide deaths in all cancers combined, as well as for each of lung, prostate, breast or CRCs. Suicide incidence and standardised mortality ratio (SMR) were estimated using SEER*Stat-8.3.2 program. Suicidal trends over time and timing from cancer diagnosis to suicide were estimated for each cancer type.

**Results:**

Among 3,640,229 cancer patients, 6,661 committed suicide. The cancer-associated suicide rate was 27.5/100,000 person years (SMR = 1.57). The highest suicide risk was observed in patients with lung cancer (SMR = 4.17) followed by CRC (SMR = 1.41), breast cancer (SMR = 1.40) and prostate cancer (SMR = 1.18).

Median time to suicide was 7 months in lung cancer, 56 months in prostate cancer, 52 months in breast cancer and 37 months in CRC (*p* < 0.001).

We noticed a decreasing trend in suicide SMR over time, which is most notable for lung cancer compared to the other three cancers. In lung cancer, suicide SMR was higher in elderly patients (70–75 years; SMR = 12), males (SMR = 8.8), Asians (SMR = 13.7), widowed patients (SMR = 11.6), undifferentiated tumours (SMR = 8.6), small-cell lung cancer (SMR = 11.2) or metastatic disease (SMR = 13.9) and in patients who refused surgery (SMR = 13).

**Conclusion:**

The cancer-associated suicide rate is nearly twice that of the general population of the United States of America. The suicide risk is highest among the patients with lung cancer, particularly elderly, widowed, male patients and patients with unfavourable tumour characteristics. The identification of high-risk patients is of extreme importance to provide proper psychological assessment, support and counselling to reduce these rates.

## Introduction

Recent advances in cancer treatment have focused on improving response, and ultimately, survival. However, a cancer patient’s emotional and psychological wellbeing is often not addressed. There is an unmet need to better understand the emotional stresses associated with the cancer diagnosis and treatment [1, 2]. In cancer, ‘survivorship’ focuses on the health and life of a person with cancer, from post-treatment until the end of life. It covers the physical, psychosocial and economic issues of cancer, beyond the diagnosis and treatment phases. It includes different issues related to the ability to get healthcare and follow-up treatment, the late effects of treatment, second cancers and quality of life. Family members, friends and caregivers are also considered part of the survivorship experience [3]. In recognition of the fact that cancer survivors have unique psycho-social stress, the National Cancer Institute created the Office of Cancer Survivorship in 1996 with the grand mission of minimising or stabilising adverse effects experienced during cancer survivorship [4]. Later, in 2002, the National Institutes of Health State-of-the-Science Conference panel issued a statement concerned with pain, depression and fatigue symptoms in cancer patients. Perhaps the most important outline was that caregivers should improve their treatment strategies to tackle the common beliefs that these symptoms are a part of the cancer experience and that tolerance is expected from the patients. The panel also recommended brief assessment tools to evaluate their subjects and validate the outcomes by adequate management [5]. The last statement by the United States Preventive Services Task Force (USPSTF) in 2016 recommended screening for depression in the general adult population, regardless of risk factors. The conclusion was based on studies that implicated depression as a leading cause for disability in the population above 15 years of age. Their recommendations were based on the fact that the benefits of screening programmes are effective enough to outweigh the harms if paired with adequate interventional programmes. Depression screening is achieved through the Patient Health Questionnaire and Hospital Anxiety and Depression Scale in adults. Other screening forms exist for specific patient populations, such as the Geriatric Depression Scale for the older population [6]. Recently, The American College of Surgeons Commission on Cancer (CoC) included survivorship care plans in cancer treatment programmes seeking CoC accreditation. Their plans included surveillance, evaluation and treatment of medical and psychosocial consequences, and the screening and promotion of healthy behaviours, according to the recommendation of the Institute of Medicine, the Lance Armstrong Foundation and the National Coalition for Cancer Survivorship [6].

Though there has been a widespread trend to screen patients for depression and suicidal thoughts, there are many variations in how these issues are handled, depending on the facility and availability of successful programmes that tend to those who screen positive. Some programmes begin at the time of diagnosis, with visits with designated staff to assess needs, and integrating other care team members as indicated (e.g. psychiatrists). Then, follow-up visits are scheduled to cover the patient after the completion of treatment. However, having a plan does not guarantee an individual’s participation in the interventions or services. Survivorship clinics, based on the type of cancer (e.g. breast or colon) or sometimes on the stage of disease, have been established in some practice settings, with educational resources made available online. Other programmes arrange group follow-up visits. The delivery of survivorship care varies widely, depending on the treatment needed, internal and external resources and recognition and/or acceptance of the importance of this aspect of health/mental healthcare [6].

In a survey based on the cancer psychosocial care matrix that included 60 cancer institutions, the existing programmes have been found to perform moderately in regards to addressing the significance of psychosocial care, identifying the need for it and referrals to appropriate services. This gap in psychosocial service capacity has been attributed to the extent of patient cooperation and the criteria upon which the provider and system work [7]. With so many variables in the system, we can identify major areas for improvement. Physicians’ awareness of the suicide risk among their recently diagnosed patients is one of the important issues.

We analysed suicide rates among cancer patients in the United States relative to the general US population, aiming to classify types of cancers according to the risk of suicide, in order to help prioritise the implementation of survivorship plans and to guide discussion and counselling of patients.

## Methods

### Data sources

The Surveillance, Epidemiology and End Results (SEER) database of the National Cancer Institute was retrospectively queried to identify cancer-associated suicide deaths in all cancers combined, as well as for each of lung, prostate, breast or colorectal cancers (CRCs). The public use version of data collected from the SEER9 registries (19 age groups) between 1973 and 2013 was used for this study, age-adjusted to the 2000 US standard based on data collected by the National Center for Health Statistics [8-10]. Suicide incidence and standardised mortality ratio (SMR) were estimated by using the SEER*Stat 8.3.2 program [10].

### Study population and inclusion criteria

In our work, we considered patients to have committed suicide if the cause of death was coded as ‘Suicide and Self-inflicted Injury (50220)’, while other cause of death values, such as ‘Accidents and Adverse Effects (50210)’, ‘Homicide and Legal Intervention (50230)’ and ‘Other Cause of Death (50300)’, were excluded. We included only patients with a single primary tumour (sequence number = 0 or 1) as patients with multiple primary tumours could not be ascribed to a single anatomic cancer site. Cases with unknown race were excluded, as well as patients for whom information was obtained solely from autopsy or death certificate, and therefore had no survival time data. We analysed all forms of malignancies to identify the overall suicide rate in the whole cancer cohort.

### Study variables

Available data in the SEER files included variables such as sex, age, race, marital status, year of diagnosis, anatomic site, extent of disease (local, regional and distant disease), date of last follow-up and vital status at last follow-up, all of which were retrieved. Although some data on psychiatric conditions, such as depression and substance abuse and comorbid medical conditions, might be important in this study, these data were not available in the SEER database. Patients who committed suicide in less than a month were coded as having a survival time of zero in the SEER registries; these were assigned a survival time of one-half month for the purpose of this study, according to a previously established, standard epidemiologic convention [11].

### Statistical analyses

Suicide rates were standardised to the sex, race and age of the persons with cancer, and the calendar year. Five-year age categories were used for standardisation. SMRs and 95%CIs were calculated by the SEER*Stat program as previously described, with SMR defined as the number of observed suicidal deaths divided by the number of expected suicidal deaths [11, 12]. Analyses of suicide relation to demographic characteristics were adjusted to age distribution in persons with cancer in the SEER registries. Analysis for SMR over the time since diagnosis was adjusted to the sex, race, age distribution and calendar year of persons with a single primary tumour in the SEER registries. The suicide rate for the general US population was obtained from National Vital Statistics Reports.

Suicide trends over time, and timing from cancer diagnosis to suicide, were estimated for each cancer type. Among lung cancer patients, the suicide SMR of different demographic, social and tumour-related factors was identified. Statistical analyses were performed with SEER*Stat 8.3.2 program (Surveillance Research Program, National Cancer Institute, Bethesda, MD) and SPSS v. 22.0 (IBM, Armonk, NY). SPSS was used to calculate the median time to suicide using the Kaplan-Meier method.

## Results

Among 3,640,229 patients diagnosed with cancer in the study period, 6,661 patients committed suicide. The cancer-associated age-, sex-, race- and calendar year-adjusted suicide rate was 27.5/100,000 person years, with the corresponding suicide rate in the US general population of 17.5/100,000 person years, which gave a SMR of 1.57 (95%CI = 1.53–1.61).

**Figure 1.**
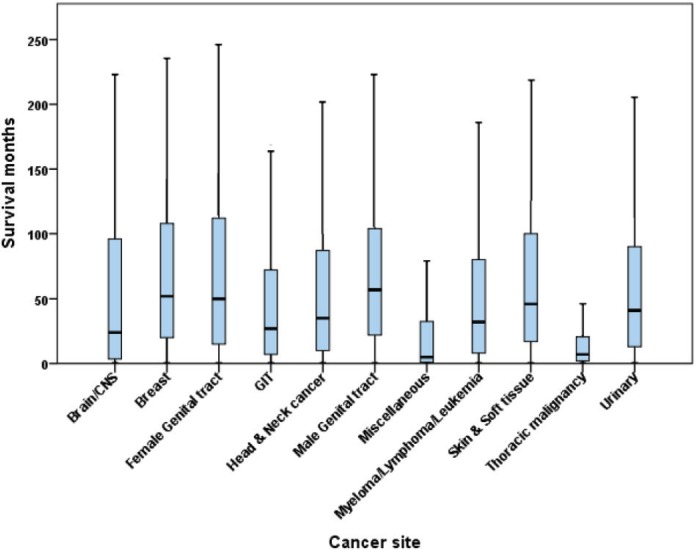
Boxplots showing the time to suicide in different types of cancer (*n* = 6,661).

The shortest median time to suicide among identified anatomical sites occurred in thoracic malignancy (median = 7 months; 95% CI = 6.24–7.77) followed by brain/central nervous system tumours (median = 24 months; 95%CI = 8.56–39.44) and gastro-intestinal tract malignancies (median = 27 months; 95%CI = 24.57–29.43), while the longest was in breast cancer (median = 52 months; 95% CI = 45.79–58.21) followed by male genital tract malignancy (including prostate cancer, cancer testis, scrotum and penis; 57 months; 95%CI = 54.11–59.89) (Figure 1).

### Suicide rates among the most common non-cutaneous malignancies

The highest suicide risk was observed in lung cancer patients [SMR = 4.17 (95%CI = 3.87–4.48)] followed by CRC (SMR = 1.41), breast cancer (SMR = 1.40) and prostate cancer (SMR = 1.18; Table 1).

The median time to suicide was 7 months from diagnosis in lung cancer, 56 months in prostate cancer, 52 months in breast cancer and 37 months in CRC (*p* < 0.001; Table 1).

Among known SEER stage cases, 48.1% of lung cancer suicides had distant stages compared to 12.1% in CRC and 5.4% and 2% in prostate and breast cancer respectively (*P* < 0.001).

### SMR trend over time

**Figure 2.**
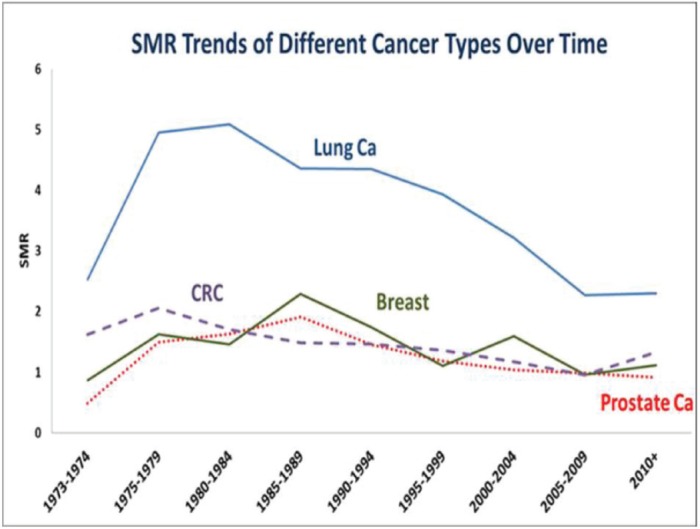
SMR trend over time.

There was a trend towards a decrease in suicide SMR over time, which is most notable for lung cancer compared to the other three cancers (Figure 2). A decline in suicide in lung cancer was noticed from 1995 forward. Prostate cancer showed the least SMR over the 40 years study period. Breast and CRC had nearly the same SMRs over the time period.

### Higher suicide rates associated variables in lung cancer

Among lung cancer patients, the suicide SMR was higher in older patients (70–75 years; SMR = 12.02), males (SMR = 8.84), Asians (SMR = 13.72), widowed patients (SMR = 11.63), patients with undifferentiated tumours (SMR = 8.57) or small-cell lung cancer (SCLC) histology (SMR = 11.16), patients presenting with metastatic disease (SMR = 13.87) and in patients who refused to receive surgical treatment (SMR = 12.88; Table 2).

### Suicide risk over time after diagnosis of lung cancer

Table 2 shows that suicide risk among persons with lung cancer was highest before the median time of suicide of 7 months compared to suicide risk in the general US population. After 7 months, notably, SMRs decrease for all variables, but remain elevated compared with that of the general US population.

Suicide SMR still was higher in males (SMR = 3.20), undifferentiated tumours (SMR = 4.81), SCLC (SMR = 5.84), metastatic disease (SMR = 5.01) and in patients who refused to receive surgical treatment (SMR = 9.10; Table 2).

## Discussion

The concern for consequences of diagnosis of cancer on the psychological well-being of patients is of primary concern. Multiple studies have addressed the issue of psychological illness in cancer patients and its effects [13–15].

However, the incidence rates for successful suicide attempts in cancer patients can be a more reflective approach to the effectiveness of methods followed to screen and counsel those patients. Last year, a study that addressed suicide in breast cancer patients who were diagnosed between the year 1973 and 2013 in the USA concluded that no significant changes in suicide rates between the four compared decades [16]. This can raise warning flags about the effectiveness of the tools implemented to counter depression and suicide in cancer patients. The relative risk for suicide among cancer patients compared to the general population can be as high as 12.6 during the first week after diagnosis, which then drops to 3.1 in the first year after diagnosis [15, 17]. However, early suicide among cancer patients seem to relate to the advanced stage of disease at diagnosis and the anatomical site [18], with biliary-pancreatic and lung cancer patients at the highest risk for SMR. Also, long disease course and unpredictable disease course causes a surge in late suicide cases, such as in breast cancer. The presence of a history of psychiatric illness can contribute to increased suicide risk and these patients should be offered counselling and medical treatment earlier in the course of disease [19]. Cancer treatments that provide the patient with improvements in their quality of life have a significant effect on the suicide rates [20, 21].

Other factors can contribute to suicides related to the specific anatomic site of the cancer. High and early suicide rates were repeatedly reported with lung and biliary-pancreatic malignancies. In breast cancer, the chronic hormonal therapy and oestrogen deprivation might be a reason for declining psychological well-being of the patient and mood disorders [22]. Similar results were concluded through a meta-analysis conducted in 2012 that included cancer patient suicide studies from 1999 to 2010 in which prostate, pancreatic and lung cancer patients were found to be at highest risk for suicide [23]. In fact, suicide among prostate cancer patients in the United States occurs at an alarming rate of 23% and anxiety among those who completed their treatment of 18.49% (CI 13.81%–24.31%), which can be another indication that some cancer treatment modalities might, in fact, play a role in affecting their psychological health [4, 15].

The effectiveness of screening tools was addressed in several studies where correlation between the presence of pessimistic thoughts and suicide was found to be of significance. A Finnish group published two studies correlating the relationship between Beck Depression Inventory results/Montgomery-Asberg Depression Rating Scale scores and negative thoughts in three groups: healthy individuals, benign breast tumour patients and malignant breast tumour patients. The authors reported that a strong correlation was found between having negative thoughts and scores of both psychiatric scoring systems, suggesting the effectiveness of these methods in identifying patients at risk [24, 25].

The 2016 USPSTF recommendations statement found that screening tools for depression in adults resulted in 17%–87% improvement in remission and/or response rates based on nine different studies, of which seven were USA-based. However, the results revealed a variation gap in the baseline population and hence, the wide range in the outcome [6].

The SMR is largely population-based and we cannot judge the USA programmes based on other populations that vary in their socio-economic structures, besides their different health programmes. The Danish cancer registry was investigated by Yousaf *et al* [26] who reported suicide SMRs of 1.7 and 1.4 among men and women, respectively, in comparison to the general Danish population. Similar conclusions were reported by Hem *et al* [27] and Björkenstam *et al* [28] for the Norwegian and Swedish national databases, respectively. In Austria, Oberaigner *et al* [29] observed an SMR of 1.86 among cancer patients in the period between 1991 and 2010, excluding non-melanoma skin cancers. In our series, the SMR was 1.57 for all cancer types where lung cancer had the highest risk of suicide. Meanwhile, cancers of the oesophagus, liver, pancreas and lung cancer had the highest SMR in Sweden according to Fang *et al* [30] and Björkenstam *et al* [28]. In Norway, males with lung cancer had the highest SMR of 4.08; however, this result was not related to chronological events of the disease [26]. We found that males had a SMR of 8.84 during the first 7 months after diagnosis compared to 3.20 thereafter. This conclusion was reported by other studies in the US such as the report by Urban *et al* [31] where lung cancer patients were found to have a higher risk for suicide, nearly five times that of the US general population. Several prior studies have reported old age and male gender to be associated with higher suicide risk [29, 30]. Asian patients reported a higher than average suicide rates, which may represent a cultural and social bias and a desire to avoid being a burden on their families.

Poor tumour prognostic features, metastatic disease and SCLC were associated with increased SMR. Nevertheless, a 52% of lung cancer suicides occurred in patients with potentially curable lung cancer stages. Anxiety and depression were at their highest rates immediately after the diagnosis of lung cancer, and this may be the reason why those patients, despite their good prognosis, have a 5.1 times higher risk of suicide within the first 3 months of diagnosis [31]. Multiple factors might be the reason for this behavioural trend, including faulty general knowledge about the prognosis of the disease, sensations of guilt, poor support systems or personal factors [30].

To reduce cancer-related suicide, patients’ understanding of cancer diagnosis and treatment options should be ensured [37]. Besides, promoting family communication combined with encouraging self-determination and participation in treatment can mitigate the social risk of suicide [37].

Overall, while advances in cancer care have been developed, greater attention must be focused on our patients’ psychological and emotional well-being. This attention may be effective in prevention if a high-risk group can be identified, as it was reported in an Australian series [32]. In addition to the importance of patients’ understanding of the cancer diagnosis and all treatment modalities, promotion of family communication combined with encouragement of self-determination and participation in treatment can mitigate the suicide risk [32–34].

The strength of this analysis is the large database query, a good resource for population-based data about cancer patients in the USA. The referral patterns and other sources of bias are less likely to affect the SEER registry, and it avoids the possible bias from hospital-based case series [31]. There are several limitations of our study. First, although some work has suggested that suicide codes are generally quite accurate [35], the literature is not conclusive as to the magnitude of misclassification bias (i.e. homicide or accidental injury may be misclassified as suicide) [36–38]. Second, the database lacks co-morbid medical and psychiatric conditions, tobacco and alcohol use, levels of psychosocial support, financial circumstances and insurance status. This distinction is necessary to further understand the real burden of suicide among cancer patients. Future assessment of factors impacting suicide may be a fertile area for further research.

## Conclusion

The cancer-associated suicide rate is nearly twice that of the general population in the USA. Suicide risk is highest among lung cancer patients, particularly older patients, widowed patients, males and patients with unfavourable tumour characteristics. It is important to identify these high-risk patients in order to provide the proper psychological assessment, support and counselling to reduce these rates. In addition to the importance of patients’ understanding of the cancer diagnosis and treatment modalities, promotion of family communication combined with the encouragement of self-determination and participation in treatment can mitigate the suicide risk.

## Conflicts of interest

The authors have no conflicts of interest to declare.

## Funding

The authors did not receive any funding for this work.

## Figures and Tables

**Table 1.** Number, SMR and median time to suicide in the four most common types of non-cutaneous cancer.

	No. of suicide cases	SMR^a^ (95%CI)	Time to suicide (Median; 95%CI)	
**Whole cancer cases**	6,661	1.57 (1.53–1.61)	33 months (31.75–34.25)	
**lung cancer patients**	739	4.17 (3.87–4.48)	7 (6.14–7.86)	*P* < 0.001
**CRC**	724	1.41 (1.31–1.52)	37 (33.51–40.49)	
**Breast cancer**	463	1.40 (1.27–1.53)	52 (45.79–58.21)	
**Prostate cancer**	1,624	1.18 (1.12–1.24)	56 (53–59)	

a Age, sex, race and year of diagnosis-adjusted calculated from Kaplan-Meier method

**Table 2.** Number, SMR and 95%CI around the median time to suicide in lung cancer of 7 months (*n* = 739).

		Suicides in ≤7 Months (*n* = 296)			Suicides in >7 Months (*n* = 443)		
		No.	%	SMR (95%CI)	No.	%	SMR (95%CI)
**Sex (*n* = 739)**	Female	30	10.14	6.92 (4.67–9.89)	53	11.96	2.55 (1.91–3.34)
	Male	266	89.86	8.84 (7.8-9.96)	390	88.04	3.20 (2.89–3.53)
**Race (*n* = 739)**	White	273	92.23	8.59 (7.6–9.67)	413	93.23	3.11 (2.82–3.43)
	Black	6	2.03	4.02 (1.48–8.76)	16	3.61	3.07 (1.75–4.98)
	Asian	17	5.74	13.72 (7.68–22.62)	14	3.16	3 (1.64–5.04)
**Marital Status (*n* = 699)**	Single	31	11.36	10.90 (7.40–15.47)	43	10.09	3.95 (2.86–5.32)
	Married	167	61.17	7.29 (6.23–8.49)	279	65.49	2.70 (2.39–3.04)
	Divorced	24	8.79	8.31 (5.33–12.37)	53	12.44	5.16 (3.87–6.75)
	Widowed	51	18.68	11.63 (8.66–15.30)	51	11.97	3.99 (2.97–5.25)
**Age (*n* = 739)**	<45	3	1.01	1.88 (0.05–10.45)	18	4.06	3.05 (1.58–5.33)
	45–49	8	2.70	6.38 (2.76–12.57)	17	3.84	2.20 (1.28–3.53)
	50–54	13	4.39	5.43 (2.89–9.28)	33	7.45	2.47 (1.70–3.47)
	55–59	31	10.47	8.39 (5.70–11.91)	58	13.09	3.15 (2.39–4.08)
	60–64	37	12.50	8.01 (5.64–11.04)	74	16.70	3.24 (2.55–4.07)
	65–69	48	16.22	8.82 (6.50–11.70)	91	20.54	3.68 (2.97–4.52)
	70–74	69	23.31	12.02 (9.35–15.21)	77	17.38	3.48 (2.75–4.35)
	75–79	43	14.53	8.29 (6–11.17)	47	10.61	2.93 (2.16–3.90)
	80–84	37	12.50	11.08 (7.80–15.27)	23	5.19	3.20 (2.03–4.80)
	85+	7	2.36	3.65 (1.47–7.52)	5	1.13	1.76 (0.57–4.12)
**Grade (*n* = 739)**	Grade I	4	1.35	3.10 (0.84–7.93)	20	4.51	1.92 (1.17–2.97)
	Grade II	25	8.45	5.67 (3.67–8.38)	69	15.58	2.39 (1.86–3.02)
	Grade III	69	23.31	7.83 (6.09–9.90)	119	26.86	2.97 (2.46–3.55)
	Grade IV	37	12.50	8.57 (6.03–11.81)	65	14.67	4.81 (3.72–6.14)
	Unknown	161	54.39	10.32 (8.78–12.04)	170	38.37	3.41 (2.91–3.96)
**Histology (*n* = 525)**	Adenocarcinoma	73	37.24	8.68 (6.81–10.92)	116	35.26	3 (2.48–3.60)
	Squamous	60	30.61	6.73 (5.14–8.66)	132	40.12	3.18 (2.66–3.77)
	Small-cell carcinoma	39	19.90	11.16 (7.94–15.26)	40	12.16	5.84 (4.17–7.95)
	Large cell carcinoma	13	6.63	6.87 (3.66–11.75)	25	7.60	3.26 (2.11–4.81)
	Carcinoid tumour	0	0.00	0 (0–12.26)	9	2.74	1.35 (0.62–2.57)
	NSCLC	11	5.61	7.93 (3.96–14.18)	7	2.13	2.05 (0.82–4.22)
**Stage (*n* = 398)**	Localised	12	6.45	2.82 (1.46–4.93)	72	33.96	1.94 (1.52–2.45)
	Regional	44	23.66	7.04 (5.11–9.45)	79	37.26	2.75 (2.18–3.43)
	Distant	130	69.89	13.87 (11.59–16.47)	61	28.77	5.01 (3.83–6.44)
**Surgical treatment (*n* = 458)**	Not recommended	117	47.95	10.47 (8.66–12.55)	76	35.51	4.02 (3.17–5.03)
	Recommended but not performed	116	47.54	11.24 (9.29–13.48)	116	54.21	6.23 (5.15–7.47)
	Recommended but refused	6	2.46	12.88 (4.73–28.04)	10	4.67	9.10 (4.36–16.74)

*NSCLC* non-small cell lung cancer
